# Editorial: Non-pharmacological Interventions in Acquired and Age-Related Cognitive Disorders

**DOI:** 10.3389/fneur.2022.878141

**Published:** 2022-04-19

**Authors:** Eliane C. Miotto

**Affiliations:** ^1^Department of Neurology, University of São Paulo, São Paulo, Brazil; ^2^Institute of Radiology, LIM-44, University of São Paulo, São Paulo, Brazil

**Keywords:** non-pharmacological interventions, cognitive training, cognitive rehabilitation, neurological disorders, aging

In the last few decades, we have seen a growing number of publications on non-pharmacological approaches to the treatment of cognitive disorders related to acquired brain lesions and aging conditions. Although most studies in this field have focused on cognitive training or rehabilitation, more recent research has explored the use of virtual reality and brain stimulation techniques such as repetitive transcranial magnetic stimulation or photobiomodulation (1). In addition, since the COVID-19 era, the number of studies adopting telerehabilitation has risen and it continues to be an important option to provide care and treatment to many patients globally (2–4).

More ecological approaches including strategies and outcome measures akin to everyday life activities have been a major challenge in this field, and research focusing on such strategies is gaining attention and priority. Examples of such strategies include face-name training and generalization of these strategies to near and far transfer tasks, the latter not directly related to the training stimuli (5). This type of approach has demonstrated strong face-validity and relevance to real life difficulties identified in these patients. This Research Topic (RT) focused on non-pharmacological interventions, comprising rehabilitation and cognitive training, and brain stimulation techniques which have been associated with cognitive and behavioral improvement across different neurological conditions. Despite some studies already being published, there is a lack of publications including a compilation of recent studies in this area. In the current RT, a total of 55 high-rank international researchers and scientists have contributed to a compilation of seven articles. Among these seven articles the readers will find four original research articles (Miotto et al.; Feger et al.; Ng et al.; Rodrigues et al.), two clinical trials (Boujut et al.; Martin et al.), and one review (Alaimo et al.).

Evidence of episodic memory improvement was found in the double-blind randomized controlled trial (RCT) of cognitive training using an ecological scenario and semantic organization strategy in stroke adults and healthy controls in comparison to an active controlled psychoeducation intervention (Miotto et al.). Behavioral gains were also observed in the transfer and generalization tasks adopting a supermarket context. Neuroimaging results showed a tendency to higher deactivation of ventromedial prefrontal areas related to the default-mode network associated with increased memory performance only in stroke patients that received cognitive training. Despite the restricted number of participants, this study indicated cognitive improvement and possible functional compensation for patients with stroke lesions after cognitive training.

Feger et al. investigated the ordering of incident difficulty across 19 instrumental activities of daily living (IADLs) in 1,277 older adults from the Advanced Cognitive Training for Independent and Vital Elderly (ACTIVE) trial. These IADLs were related to seven task categories including meal preparation, housework chores, use of phone, shopping, traveling, finance, and health care managing. Participants were followed up for 10 years. Housework, managing health care, and phone use were the first task categories to become difficult independently of group allocation (cognitive training or control group). The authors suggested that since the intervention with cognitive training focused on a unique area of cognition, it did not have an effect ordering in terms of IADL problems specially due to the fact that IADL activities involve abilities and multiple cognitive domain integration.

Ng et al. compared the efficacy of computerized cognitive training (CCT) in improving cognition and self-reported world experience in older (50–80) compared to younger (18–49) adults based on a previously published study using the Lumosity program. The authors found similar gains across the participants with overall effects of CCT of small to medium size. Using Repetitive Transcranial Magnetic Stimulation (rTMS), Rodrigues et al. investigated the effects of 10 sessions, each session for 20 min, using 10-Hz rTMS on the left dorsolateral prefrontal cortex for treatment of anxiety, depression, and executive functions in moderate to severe chronic traumatic brain injury patients. All patients included in the research finished the protocol and *post-hoc* analysis was carried out. The main findings of their study indicated an improvement in depression and executive functions after the 10 sessions, although no time and group interaction was found. In addition, there was no interaction between patient group and treatment related to anxiety scores. Furthermore, they found no adverse effects associated with the rTMS intervention. These results are interesting and future research comparing follow-up sessions with and without boost sessions can perhaps help to expand these findings further.

Martin et al. investigated photobiomodulation (PBM) in red/near-infrared (NIR) wavelengths applied transcranially in Gulf War Illness (GWI) to enhance cognition and health-related symptoms. In their blinded and randomized sham-controlled trial with sham or real red/NIR light emitting diodes (LED), 48 volunteers with GWI were included. They underwent 15 half an hour sessions of transcranial LED treatment twice a week for a period of 7.5 weeks. The authors used cognitive, health, and physical outcome measures and found improvement on the Digit Span test and SF-36V Plus and Physical Component Score for the real group in comparison to the sham group. In particular, participants that had increased post-traumatic stress disorder (PTSD) symptoms before intervention who underwent real treatment, showed a PTSD reduction after 1 month. Despite the reduced sample, future transcranial LED home treatment devices are warranted.

In another clinical trial, Boujut et al. investigated the Attentional Control Training in Older People (ACTOP) of updating and inhibition training efficacy and transfer in 90 cognitively healthy older adults. Participants received 12 half an hour sessions of either an active control quiz game of general knowledge or computerized training sessions for updating using tasks such as N-back and for inhibition using Stroop-like tasks. In addition, 30 younger adults underwent working memory (WM) and proximal transfer tasks without training. Authors demonstrated an improvement for both the updating and inhibition training groups and, on the WM, proximal transfer measures. However, younger adults showed better performance in comparison to older ones on all transfer tasks before training. The authors pointed out that although a general improvement was found in older adults on all transfer tasks, the training interventions including updating and inhibition did not show further enhancements when compared to the active control condition. They discuss this issue suggesting that the effectiveness of process-based training may not impact transfer tasks.

Alaimo et al. presented in their review a summary of protocols with remotely controlled cognitive training and individualized feedback in healthy older participants or those with subjective memory complaints provided by their therapist. The authors identified eight papers including five with healthy older adults and three volunteers with subjective memory complaints. The majority indicated improvement after cognitive telerehabilitation particularly on memory, attention, working memory, executive functions, and language functions. In addition, they found a reduction of mood symptoms such as anxiety and depression. There were also decreased subjective memory problems reported. The authors indicated that cognitive telerehabilitation interventions may provide an effective alternative to face-to-face intervention and highlight the value of cognitive interventions in people with subjective memory complaints or subjective cognitive decline.

Over the last few decades, we have seen the emergence of innovative and technological approaches in the non-pharmacological rehabilitation field. The current RT contributes to a broader understanding of non-pharmacological interventions in patients with cognitive disorders with emphasis on cognitive training, rehabilitation, and brain stimulation techniques. The present findings have also pointed to important topics that will be relevant for future research including methodological issues such as individual techniques vs. multidomain approaches, tasks and brain mechanisms facilitating transfer-related effects, inclusion of biomarkers and neuroimaging methods to increase appropriate population inclusion criteria, and understanding of the neural correlates associated with these interventions.

## Author Contributions

The author confirms being the sole contributor of this work and has approved it for publication.

## Conflict of Interest

The author declares that the research was conducted in the absence of any commercial or financial relationships that could be construed as a potential conflict of interest.

## Publisher's Note

All claims expressed in this article are solely those of the authors and do not necessarily represent those of their affiliated organizations, or those of the publisher, the editors and the reviewers. Any product that may be evaluated in this article, or claim that may be made by its manufacturer, is not guaranteed or endorsed by the publisher.

## References

[1] Miotto EC Batista AX Simon SS Hampstead BM. Neurophysiologic and cognitive changes arising from cognitive training interventions in persons with mild cognitive impairment: a systematic review. Neural Plast. (2018) 2018:7301530. 10.1155/2018/7301530 30766600 PMC6350540

[2] Mantovani E Zucchella C Bottiroli S Federico A Giugno R Sandrini G. Telemedicine and virtual reality for cognitive rehabilitation: a roadmap for the COVID-19 pandemic. Front Neurol. (2020). 11:926. 10.3389/fneur.2020.00926 33041963 PMC7522345

[3] Maggio MG De Luca R Manuli A Calabrò RS. The five ‘W'of cognitive telerehabilitation in the COVID-19 era. Expert Rev Med Dev. (2020) 17:473–5. 10.1080/17434440.2020.1776607 32476504

[4] Bernini S Stasolla F Panzarasa S Quaglini S Sinforiani E Sandrini G. Cognitive telerehabilitation for older adults with neurodegenerative diseases in the COVID-19 era: a perspective study. Front Neurol. (2021) 11:623933. 10.3389/fneur.2020.623933 33519704 PMC7840692

[5] Batista AX Bazán PR Conforto AB Martin MDGM Simon S Hampstead B. Effects of mnemonic strategy training on brain activity and cognitive functioning of left-hemisphere ischemic stroke patients. Neural Plast. (2019) 2019:4172569. 10.1155/2019/4172569 31210761 PMC6532294

